# Ultrasonic treatment of *Dendrobium officinale* polysaccharide enhances antioxidant and anti‐inflammatory activity in a mouse D‐galactose‐induced aging model

**DOI:** 10.1002/fsn3.2867

**Published:** 2022-04-01

**Authors:** Wenhui Chu, Pan Wang, Zhe Ma, Lin Peng, Zongmin Wang, Zilin Chen

**Affiliations:** ^1^ School of Life Science Taizhou University Taizhou China; ^2^ Traditional Chinese Medicine Industry Development and Promotion Center of Pan'an County Pan'an China; ^3^ 91620 School of Agriculture and Food Engineering Shandong University of Technology Zibo China

**Keywords:** anti‐inflammatory activity, antioxidant activity, *Dendrobium officinale*, D‐galactose, polysaccharide

## Abstract

Utilization of the biological macromolecule *Dendrobium officinale* polysaccharide (DOP) as a functional ingredient is limited by its high intrinsic viscosity and molecular weight. The goal of the present study was to improve rheological properties of DOP by ultrasonic treatment. Such a treatment resulted in the degradation of DOP and consequent reduction of rheological properties. Among DOP samples treated with ultrasonication at low (L), medium (M), and high (H) power intensities (25, 50, 75 w/cm^2^), M‐DOP displayed the highest reactive oxygen species (ROS) and reactive nitrogen species (RNS) radical scavenging activity in vitro. In a mouse D‐galactose (D‐Gal)‐induced aging model, M‐DOP significantly increased activities of antioxidant enzymes and reduced levels of pro‐inflammatory cytokines in liver. Real‐time polymerase chain reaction (RT‐PCR) analysis indicated that M‐DOP upregulated messenger RNA (mRNA) expression of anti‐inflammatory/antioxidant proteins such as Nrf2 (nuclear factor erythroid 2‐related factor), hemeoxygenase‐1 (HO‐1), and NAD(P)H:quinone oxidoreductase (NQO1) in liver. In summary, M‐DOP displayed a strong radical scavenging activity in vitro, and ameliorated liver injury in the mouse aging model through the promotion of Nrf2/HO‐1/NQO1 signaling pathway.

## Graphical abstract

After the ultrasonic treatment, the rheological property and structural properties of *Dendrobium officinale* polysaccharide were improved. Ultrasonic treatment also improved the antioxidant and antiaging activity in D‐galactose (D‐Gal) mice. Treated polysaccharide reduced liver injury in D‐galactose mice through the promotion of Nrf2/HO‐1/NQO1 (nuclear factor erythroid 2‐related factor/hemeoxygenase‐1/NAD(P)H:quinone oxidoreductase) signaling pathway.

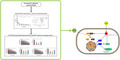

## 1 INTRODUCTION

The genus *Dendrobium* is the largest genus in the Orchidaceae family (>1100 species), and many of the species are part of the “traditional medicine” used for centuries in China and other Asian countries. In particular, extracts from the stem of medicinal species *D. officinale* display several beneficial pharmacological effects, *e.g*., improvement of body fluid composition, gastric tonicity, and immune system function (Xing et al., 2013). The stem can be chewed intact or added to other foods. During the past two decades, *D. officinale* compounds have received increasing research attention and have been commercially developed as ingredients of functional beverages, capsules, and powders.

The major bioactive component of *D. officinale* is a water‐soluble polysaccharide macromolecule. Polysaccharides derived from various *Dendrobium* species are characterized by high intrinsic viscosity. Pan et al. (2014) found that viscosity of polysaccharides from four *Dendrobium* species ranged from 35 to 127 cm^3^/g. Molecular weight (Mw) of the four polysaccharides ranged from 197 to 378 kDa, and high Mw appeared to be associated with high viscosity (He et al., 2018). The high Mw and viscosity of *D. officinale* polysaccharide (DOP) make it useful as a gelling or thickening agent in food industries. On the other hand, relatively low Mw and viscosity are preferable for bioactive components in functional foods or beverages. Numerous methods have been applied for depolymerization of polysaccharides, including chemical treatment (Mzoughi et al., 2017), enzyme hydrolysis (Yan et al., 2018), ultrasonic treatment (Qiu et al., 2019), and γ‐irradiation (Wang et al., 2017). Among these, ultrasonic treatment has several advantages: it is rapid, mild, environmentally friendly, and does not involve toxic reagents. In some cases, immunoregulatory (Yao et al., 2015), antioxidant, and/ or antitumor activity (Yan et al., 2016) of polysaccharides is enhanced by ultrasonic treatment. In the present study, rheological properties (*i.e*., deformation or flow behaviors in response to applied forces) of DOP were improved by ultrasonic treatment.

Reactive oxygen species (ROS), in their normal function as signaling molecules, are involved in the resumption of meiosis following meiotic arrest, and in the activation of apoptotic pathways (Agarwal et al., 2005). However, in unbalanced states in vivo, ROS may be overproduced and lead to oxidative damage, *e.g*., lipid peroxidation (Fan & Li, 2014), cross‐linking and degeneration of biomacromolecules by malondialdehyde (MDA) (Hipkiss et al., 1998), and inflammatory response (Byun et al., 2017). Accumulation of ROS and other free radicals is a major mechanism in aging processes and contributes to brain aging and senile dementia (Salmon et al., 2010).

D‐galactose (D‐Gal), a reducing sugar, is converted to glucose by galactose (Gal)‐1‐phosphate uridyltransferase and galactokinase at physiological concentration. Excessive D‐Gal levels resulted in disordered cellular metabolism, alteration of oxidase activity, and production of oxidative products (Li et al., 2016). The natural aging process was recapitulated in a D‐Gal‐induced mouse model based on a free radical theory of aging (Çoban et al., 2015). Besides causing oxidative stress, excessive accumulated D‐Gal reacts with free amino acid groups of proteins or peptides, with consequent formation of advanced glycation end‐products (AGEs) (Zhou et al., 2015). Numerous studies have demonstrated that ROS and AGEs are key factors contributing to aging processes and related diseases (*e.g*., liver damage, nephritis, Alzheimer's disease), and other diseases (Palma‐Duran et al., 2018; Saleh et al., 2019). D‐Gal‐induced aging models are therefore used frequently in antiaging pharmacological research.

We applied the ultrasonic treatment to improve the rheological properties of extracted DOP and evaluated structural properties (monosaccharide composition, chemical composition, Mw) during treatment. Radical scavenging activity was examined in vitro to identify the active fraction of DOP following ultrasonic treatment. Biochemical indexes and pro‐inflammatory cytokines in serum and liver following DOP treatment were analyzed in a mouse D‐Gal‐induced aging model to clarify relationships between structure and biological activity of the polysaccharide.

## 2 MATERIALS AND METHODS

### 2.1 Materials and reagents

*Dendrobium officinale* collected at 3 years after greenhouse planting was purchased at a Chinese herbal medicine market and identified by Associate Professor and senior lab master Tong Chen (School of Life Science, Taizhou University). Dextran with various Mw values, 2,2‐diphenyl‐1‐picrylhydrazyl (DPPH), 1,10‐phenanthroline, nitrotetrazolium blue chloride, phenazine methosulfate, NADH, sodium nitroprusside, and D‐Gal were from Sigma‐Aldrich. Other reagents used were of analytical grade.

### 2.2 *D. officinale* polysaccharide (DOP) extraction

*Dendrobium officinale* stems were dried at 105°C for at least 12 h until reaching a constant weight and then ground to powder (50‐mesh sieve). The powder was extracted in 1000 ml boiling water for 3 h. Extract supernatant was collected by centrifugation (5000 × *g*, 10 min), and residual powder was subjected to the same extraction process twice. Combined supernatants were added with ethanol (4× volume). Ethanol residue was removed, and precipitates were deproteinized by Sevage method and concentrated by lyophilization to obtain DOP.

### 2.3 Depolymerization of DOP by ultrasonic treatment

An ultrasound generator (Jiangsu Bode Ultrasound Equipment Co.) was used for ultrasonic treatment. Twenty milliliters of DOP solution (1 mg/ml) was treated with 20 kHz/750 W, and a bottle containing the solution was kept in ice water to avoid overheating. Amplitude parameters 20%, 40%, and 60% were set using the control panel. Ultrasound power intensities (power per unit area of probe tip) corresponding to these three amplitudes were, respectively, 25, 50, and 75 w/cm^2^, based on the formula described previously (Li et al., 2004). Following ultrasonic treatment, DOP was separated by ultrafiltration membrane (Mw cut‐off 30 kDa), concentrated by lyophilization, and subjected to analysis of structural properties and biological activities.

### 2.4 Intrinsic viscosity analysis

*Dendrobium officinale* polysaccharide was dissolved to various concentrations (1.0, 1.25, 1.5, 2.0, 2.5 mg/ml), and viscosity of the solutions was measured by Ubbelohde‐type viscometer (Hangzhou Zhuoxiang Technology Co.) at 25°C. Each solution was centrifuged (5000 × *g*, 10 min), filtered using 0.45‐μm membrane, and elapsed time going through the viscometer was recorded. Intrinsic viscosity was defined as the intercept of ln(ηr/C) and C curves, where ηr = relative viscosity and C = concentration (Yan et al., 2009).

### 2.5 Chemical composition analysis

Chemical composition analysis was performed to determine contents of carbohydrates (Dubois et al., 1956), proteins (Bradford, 1976), and sulfate radicals (Dodgson & Price, 1962) in DOP, using the methods described in these studies.

### 2.6 Monosaccharide composition analysis

Monosaccharide composition of DOP was analyzed by gas chromatography (GC), as described in our previous report (Peng et al., 2015). In brief, 20 mg of polysaccharide was hydrolyzed by H_2_SO_4_ and neutralized by BaCO_3_, the obtained samples were reacted with pyridine and acetic anhydride, and the solution of monosaccharide derivatives was analyzed by GC. Inositol was used as internal standard for the calculation of mole percentage.

### 2.7 Molecular weight analysis

Mw of DOP was measured by high‐performance gel filtration chromatography (HPGFC) with Ultrahydrogel Linear Column, and elution by 0.1 M NaNO_3_ at a flow rate 0.9 ml/min. DOP solution (20 μl; 0.1%, w/v) was injected, and Mw was recalibrated using dextran with various Mw values.

### 2.8 Fourier‐transform infrared spectroscopy (FTIR) analysis

*Dendrobium officinale* polysaccharide samples were mixed with potassium bromide (KBr) powder (1:100) and compacted into pellets, which were analyzed by Tensor 27 FT‐IR spectrometer (Bruker; Pittcon 2019 Expo). FTIR spectra were recorded in the range of 400–4000 cm^−1^, the baseline was adjusted using the computer program supplied with the instrument, and the FTIR profile was plotted using the software program Origin 8.

### 2.9 Antioxidant activities

#### 2.9.1 Hydroxyl radical scavenging activity

This type of activity was analyzed by the methods described previously (Wang et al., 2013). In brief, 2 ml DOP solution (various concentrations) was mixed with 1 ml 1,10‐phenanthroline (1.865 mM) and 1 ml FeSO_4_.7H_2_O solution (1.865 mM). The reaction was initiated by adding 1 ml H_2_O_2_ (0.03% v/v) and incubated for 1 h at 37°C. Control sample was a reaction mixture without addition of DOP solution, and blank sample was a mixture without addition of H_2_O_2_. Absorbance at 536 nm (A) was measured and applied in the formula:

$$\text{Hydroxyl radical scavenging activity}\;\left(\%\right)=\frac{A_{\text{sample}}‐A_{\text{control}}}{A_{\text{blank}}‐A_{\text{control}}}\times 100$$

#### 2.9.2 Superoxide anion radical scavenging activity

This activity was also determined as described by Wang et al. (2013). One milliliter of DOP solution (various concentrations) was mixed with 1 ml nitrotetrazolium blue chloride (2.52 mM) and 1 ml NADH (264 mM), added with 1 ml phenazine methosulfate (0.12 mg/ml), and the reaction mixture was incubated for 5 min at 25°C. Control sample was a reaction mixture without addition of DOP. Absorbance at 560 nm was measured and applied in the formula:

$$\text{Superoxide anion radical scavenging activity}\;\left(\%\right)=\frac{A_{\text{control}}‐A_{\text{sample}}}{A_{\text{control}}}\times 100$$

#### 2.9.3 Nitric oxide (NO) radical scavenging activity

This activity was determined as described previously (Yen et al., 2001). As much as 0.25 ml DOP solution (various concentrations) was mixed with 0.25 ml sodium nitroprusside (10 mM, dissolved in phosphate‐buffered saline (PBS) solution). The reaction mixture was incubated for 2 h at 25°C and then mixed with an equal volume of the Griess reagent (Sigma) and incubated for 10 min at room temperature. Control sample was a reaction mixture without addition of DOP. Absorbance at 540 nm was measured and applied in the formula:

$$\text{Nitric oxide radical scavenging activity (}\%)=\frac{A_{\text{control}}‐A_{\text{sample}}}{A_{\text{control}}}\times 100$$

### 2.10 Animal experiments

Forty Kunming mice (7‐week‐old, male, ~20 g) (SCXK [Hu] 2007‐0005) were obtained from Silaike Laboratory Animal Co. (Shanghai). Mice were maintained at 25°C, 60% relative humidity, automated 12 h light/12 h dark cycle. Following a 1‐week period of adaptation, the 40 mice were divided randomly into six groups: control group (Con); model group (treated with D‐Gal but not M‐DOP); low‐dose M‐DOP (M‐DOP‐L; 250 mg/kg body weight [BW]/day); medium‐dose M‐DOP (M‐DOP‐M; 500 mg/kg BW/day); high‐dose M‐DOP (M‐DOP‐H; 1000 mg/kg BW/day); positive group (VE; 150 mg/kg BW/day). The Con group was administered 0.9% saline w/w, while the other five groups were intraperitoneally administered D‐Gal (200 mg/kg BW/day) for 8 weeks. The four experimental groups (M‐DOP‐L, ‐M, ‐H and VE) were administered M‐DOP by oral gavage 1 h after D‐Gal injection. At the conclusion of experiments, mice were fasted for 24 h and killed by cervical dislocation. Blood was collected from abdominal aorta, transferred to a nonanticoagulated blood collection tube, and centrifuged (3000 rpm, 10 min, 4°C). The samples were stored at −80°C for further analysis. All animal experiments were conducted in accordance with established legislative and ethical guidelines of relevant regulatory agencies in China.

### 2.11 Determination of body weight, biochemical indexes, and cytokine levels

BWs were recorded (simultaneously for all mice) every 2 weeks. Collected liver tissues (defined amount) were homogenized, and supernatants were analyzed. Serum biochemical indexes (alanine aminotransferase [ALT], aspartate aminotransferase [ASP], alkaline phosphatase [ALP], and creatinine [CREA]) were evaluated using detection kits (Nanjing Jiancheng Technology Co. Ltd.) as per manufacturer's instructions. Levels or activities in liver of malondialdehyde (MDA), glutathione (GSH), superoxide dismutase (SOD), catalase (CAT), and glutathione peroxidase (GSH‐Px) were also evaluated with kits (Nanjing Jiancheng). Levels of pro‐inflammatory cytokines (interleukin 1β [IL‐1β], interleukin 6 [IL‐6]) in liver were determined by an enzyme‐linked immunosorbent assay (ELISA) kit (Sigma). Nitric oxide (NO) level in liver was determined by mixing with Griess reagent, with sodium nitrite as standard.

### 2.12 Real‐time quantitative PCR (RT‐qPCR)

Livers from the six groups were collected, and total RNA was extracted using RNA isolation kit (Generay Biotech Co.) as per manufacturer's instructions. Complementary DNA (cDNA) was reverse‐transcribed from RNA using the RT‐PCR kit (Generay). The cDNA template was mixed with SYBR Green PCR Master Mix and primers are listed in Table S1 (Appendix S1). The reaction was performed at 95°C for 60 s, followed by 40 cycles of denaturation at 95°C for 15 s, annealing at 58°C for 30 s, and extension at 72°C for 35 s. Glyceraldehyde‐3‐phosphate dehydrogenase (GAPDH) was used as internal reference, and the relative gene expression was calculated by the 2^−ΔΔCt^ method.

### 2.13 Statistical analysis

Animal experiments were conducted using eight replicates, and other experiments with three replicates. Data were expressed as mean ± SD. Comparisons between two groups were made by one‐way analysis of variance (ANOVA) using GraphPad Prism software program, V. 7.0. Differences with *p* < .05 were considered significant.

## 3 RESULTS AND DISCUSSION

### 3.1 Structural characterization of DOP subjected to three ultrasonic treatments

The high intrinsic viscosity of DOP resulting from high Mw limits its potential application as a functional food ingredient. We depolymerized DOP by ultrasonic treatment (frequency 20 kHz, power 750 w). DOPs treated with three power intensities (25, 50, 75 w/cm^2^), respectively, termed L‐, M‐, and H‐DOP, all displayed a sharp reduction of intrinsic viscosity after 20 min treatment, and a fairly constant level thereafter (Figure S1). Structural differences among L‐, M‐, and H‐DOP were investigated by FTIR, monosaccharide composition, and chemical composition analyses, as described below.

Structural properties were evaluated for L‐, M‐, and H‐DOP treated with or without ultrasonication for 40 min, and for native DOP (N‐DOP). Peaks in FTIR spectra were identified as shown in Figure S2, based on a previous study (Huang et al., 2016). The spectra displayed broad characteristic peaks at 3420 cm^−1^ for hydroxyl stretching vibration, and at 2926 cm^−1^ for C–H stretching vibration. Peaks at ~1735 and 1250 cm^−1^ were attributed to carboxyl and C–O bonds of carboxylic acid (COOH). Absorption peaks at 1064 and 809 cm^−1^ indicated the presence of pyranose and of β‐D‐mannose glucosidic linkage, respectively. Major structural properties of the four DOPs were similar; however, carboxyl and C–O bonds of COOH were more strongly affected by ultrasonic treatment for H‐DOP than for L‐, M‐, or N‐DOP.

Mw was the highest (617 kDa) for N‐DOP and lowest (72.8 kDa) for H‐DOP (Table 1). The low value for H‐DOP is attributable to ultrasonic depolymerization which disrupts the molecular bond of polysaccharide (Zhu et al., 2014). Mw values of M‐ and H‐DOP were significantly lower than those of N‐ and L‐DOP, reflecting the depolymerization of polysaccharide by ultrasound. Within a certain range, the intrinsic viscosity of DOPs is evidently associated with Mw. However, below this range, reduction of intrinsic viscosity cannot be attributed simply to reduced Mw.

**TABLE 1:** Molecular weight (Mw) and monosaccharide composition of *Dendrobium officinale* polysaccharide (DOP) samples

Sample	Mw (kDa)	Monosaccharide composition (mole ratio)					
		Ara	Gal	Glc	Man	Rha	Xyl
N‐DOP	617 ± 17.23^a^	0.37	0.43	1.00	0.11	0.04	0.02
L‐DOP	266 ± 10.3^b^	0.41	0.45	1.00	0.13	–	–
M‐DOP	75.41 ± 23.12^c^	0.38	0.40	1.00	0.15	0.02	–
H‐DOP	72.80 ± 14.83^c^	0.39	0.42	1.00	0.12	–	–

Note

N‐DOP indicates the crude polysaccharide with no ultrasound treatment. L‐, M‐, and H‐DOP indicate different ultrasound power intensities: respectively, 25, 50, and 75 w/cm^2^. Different letters (a, b, c) indicate significant differences (*p* < .05).

–: No monosaccharide was detected.

Monosaccharide composition analysis of DOPs revealed six major monosaccharides (arabinose, Gal, glucose [Glc], mannose, rhamnose, xylose) (Table 1). Among these, mole ratio was higher for Glc than for the other five, consistently with previous findings by J.P. Luo's group (Pan et al., 2014). Ultrasonic treatment of the DOPs had no effect on monosaccharide composition or mole ratio, indicating that it does not alter polysaccharide chains or molecular bonds.

Contents of carbohydrates, proteins, and sulfate radicals for the four DOPs before and after ultrasonic treatment are shown in Table S2. Protein and sulfate radical contents were highest for N‐DOP without ultrasonication. In general, ultrasonic treatment increased carbohydrate content (*p* < .05) and decreased protein and sulfate radical contents (*p* < .05). Sulfate radical content was lower for H‐DOP than for L‐ or M‐DOP. S. Huang's group observed previously that protein and sulfate radical contents of DOP samples varied depending on the extraction method (He et al., 2018). We observed that ultrasound power intensity is also a key factor affecting DOP chemical composition.

Analysis of structural properties showed that ultrasonic treatment of DOPs primarily affected carboxyl and C–O bonds, and protein and sulfate radical contents. Primary structure of DOP treated with various ultrasound power intensities (L‐, M‐, H‐DOP) may not differ notably from that of N‐DOP.

### 3.2 Antioxidant activity of DOPs

**FIGURE 1:**
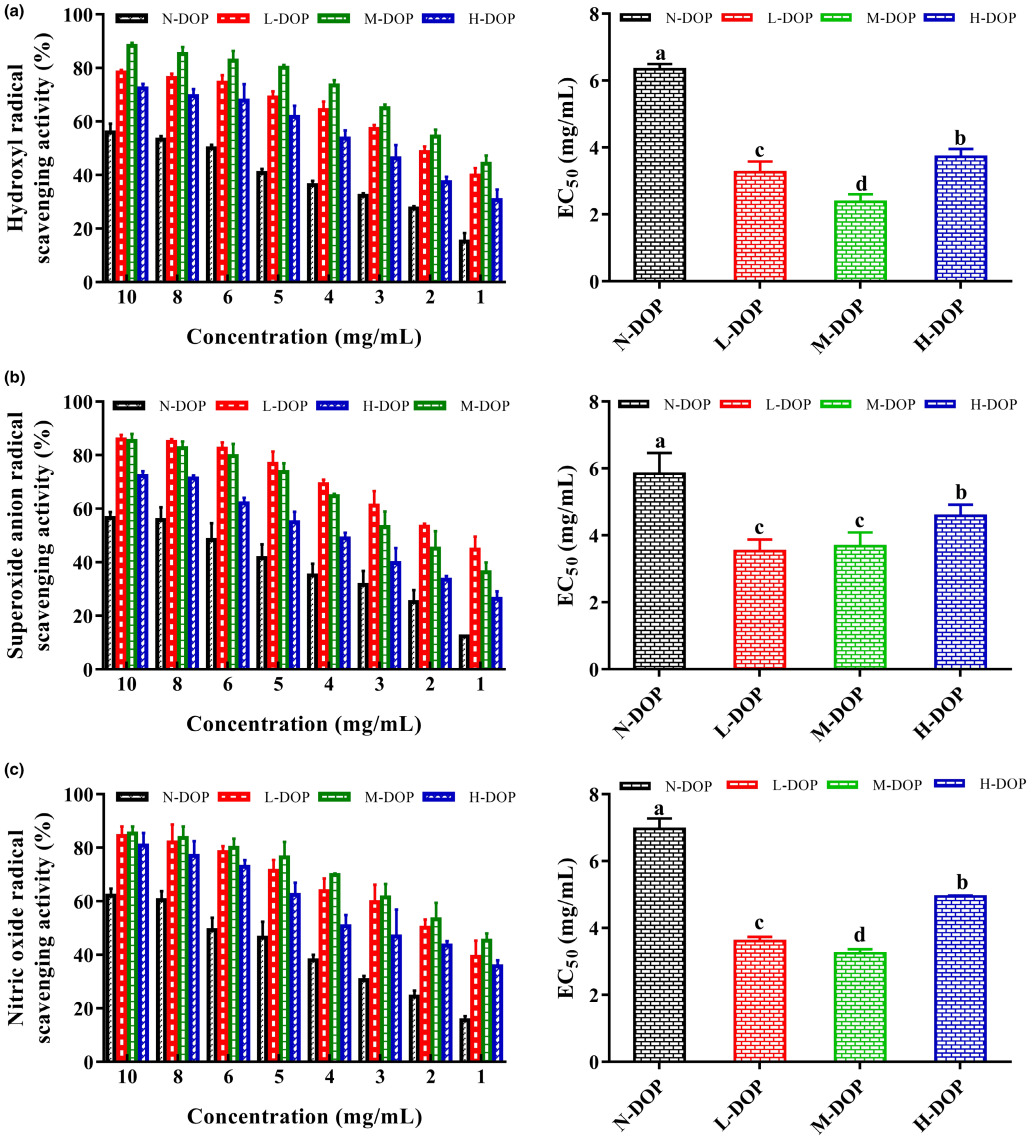
In vitro scavenging activity of *D. officinale* polysaccharide (DOP) with or without ultrasonic treatment on (a) hydroxyl, (b) superoxide anion, and (c) nitric oxide (NO) radicals. Different letters (a, b, c, d) indicate significant differences (*p* < .05)

In normal cell metabolism, dynamic redox equilibrium is maintained by the elimination of excess ROS (*e.g*., hydroxyl radical, superoxide anion radical) by antioxidants (GSH, NADPH) and detoxification enzymes (catalase [CAT], superoxide dismutase [SOD], glutathione peroxidase [GSH‐Px]) (Yang et al., 2018). Oxidative stress, resulting from insufficient levels of antioxidants and detoxification enzymes, leads to lipid peroxidation and protein oxidation, and promotes tissue injury (Naik & Dixit, 2011; Salzano et al., 2014). Data on hydroxyl radical scavenging activity (Figure 1a) indicate strong activity for all four samples at tested concentrations, and correlation of activity with concentration. Among the four samples, M‐DOP had the highest hydroxyl radical scavenging activity and the lowest EC_50_ (2.28 mg/ml). Superoxide anion scavenging EC_50_ values for L‐, H‐, and N‐DOP were, respectively, 3.53, 3.68, and 5.84 mg/ml (Figure 1b). All four samples displayed NO radical scavenging activities, which were correlated with concentrations (Figure 1c). The EC_50_ value was significantly lower for M‐DOP (3.24 mg/ml) than for the other samples, indicating that M‐DOP had the strongest NO radical scavenging activity.

Mw is an important determinant of antioxidant activity. Previous studies demonstrated that antioxidant activity varied depending on Mw for various polysaccharides, *e.g*., in *Lycium barbarum* (Liu et al., 2021), *Codium cylindricum* (Yan et al., 2021), and fucoidan (Hou et al., 2012). Reduction of Mw for such polysaccharides enhanced antioxidant activities, including radical scavenging activity and reducing power. Q. Li's group proposed that reduction of Mw resulted in the exposure of more reducing ends for such polysaccharides (Yu et al., 2021). Thus, the high ROS and reactive nitrogen species (RNS) radical scavenging activities we observed for M‐DOP may have been due to decreased Mw and increased exposure of reducing ends.

### 3.3 Liver tissue histopathology

**FIGURE 2:**
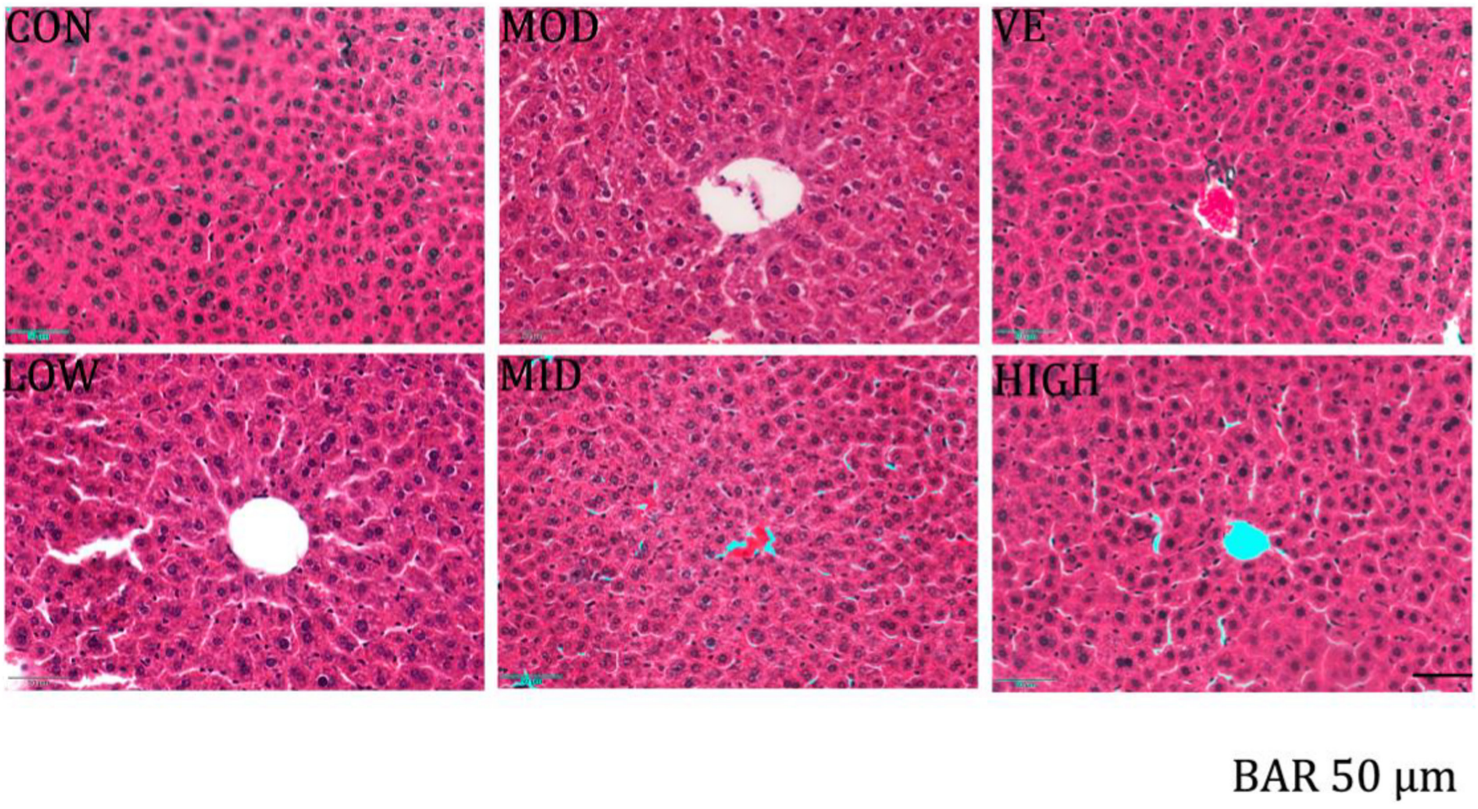
Effect of M‐DOP on liver histology in D‐galactose (D‐Gal)‐injected mice, revealed by hematoxylin–eosin (HE) staining. CON, control group; MOD, model group; VE, positive group; LOW, D‐Gal + 250 mg/kg M‐DOP; MID, D‐Gal + 500 mg/kg M‐DOP; HIGH, D‐Gal + 1000 mg/kg M‐DOP. Scale bar: 50 µm

Excessive D‐Gal was previously shown to induce liver damage in an aging mouse model (Chen et al., 2020; Li et al., 2019). Similarly, we observed D‐Gal‐induced histological changes in liver tissues in our mouse model (Figure 2). Cell architecture of the liver tissue in the normal group reflected normal hepatocytes with central veins. In the model group, D‐Gal injection resulted in a major liver damage, including hepatocellular hydropic degeneration, necrosis, and binucleation. Such damage was gradually ameliorated by the M‐DOP administration in M‐DOP and VE groups. At M‐DOP dose of 500 mg/kg, VE and M‐DOP groups were similar in terms of liver physiological status, including structural changes and inflammatory cell infiltration. These findings indicate that hepatic pathological changes caused by excessive D‐Gal were ameliorated by M‐DOP treatment.

### 3.4 Effect of DOP on the liver function in mouse D‐Gal‐induced aging model

In vitro experiments revealed strong promotion by M‐DOP of ROS and RNS radical scavenging activity (Figure 1). We further investigated effects of M‐DOP treatment on antioxidant and antiaging activity in vivo using a mouse D‐Gal‐induced aging model. During an 8‐week feeding period, all five experimental groups showed increased BW (Figure S3). At the end of the feeding period, BWs were notably higher for control group than for model group. BW reduction resulting from D‐Gal administration was offset by M‐DOP treatment.

Declining function of organs in the commonly used mouse D‐Gal‐induced aging model simulates that in natural aging (Chen et al., 2010). In the mouse model, excessive D‐Gal causes accumulation of ROS and formation of advanced glycation end‐products (AGEs), which lead to oxidative damage (Aydin et al., 2011). Liver health status can be assessed by measuring the serum content of various enzymes and biochemicals. We evaluated the degree of hepatic injury by measuring serum activities of ALT, ASP, and ALP, and CREA level. All these parameters were significantly (*p* < .05) higher in model than in control group (Figure S4), and were reduced in M‐DOP groups relative to model group. These findings clearly indicate a protective effect of M‐DOP against D‐Gal‐induced liver injury in this model.

**FIGURE 3:**
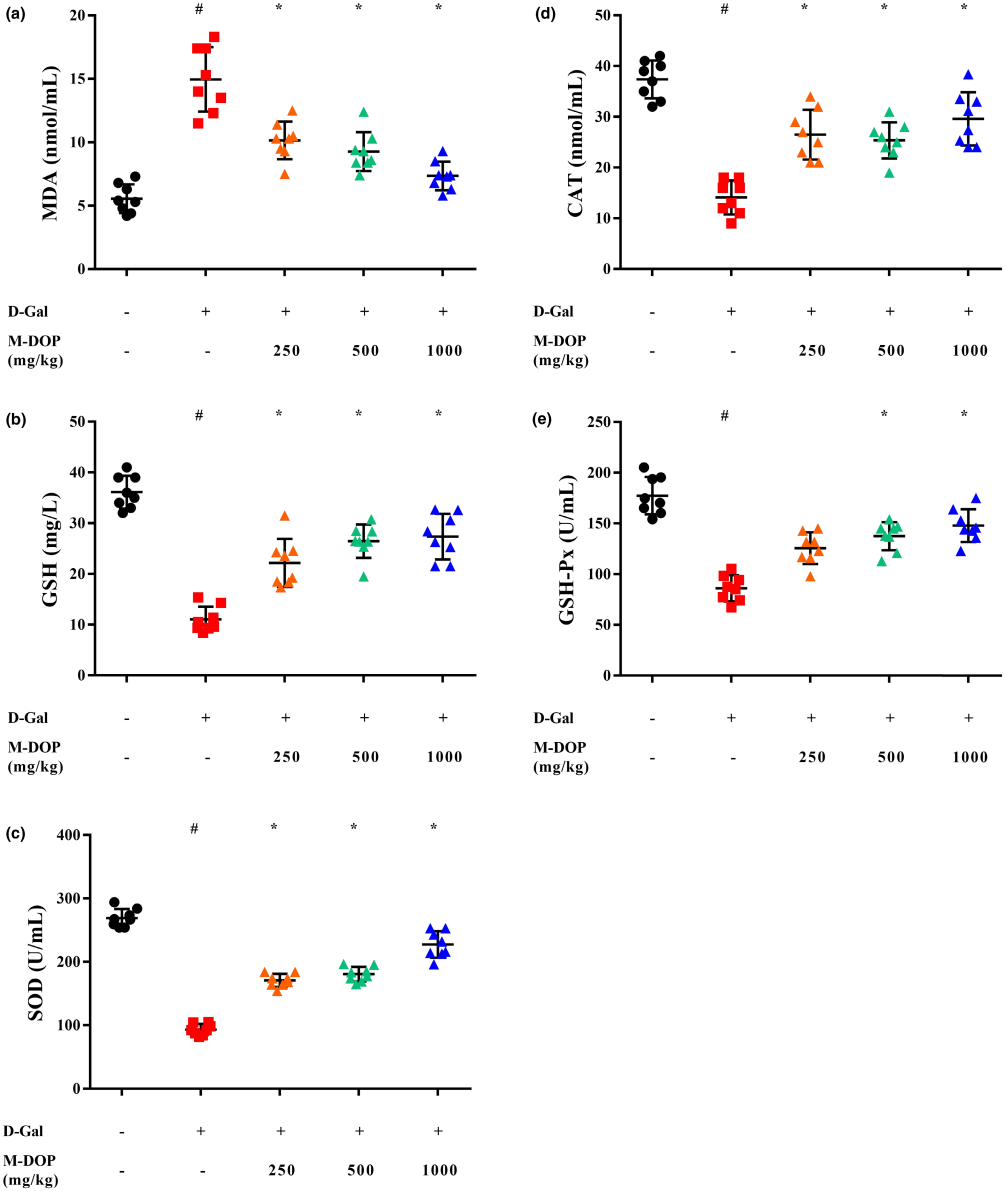
Effects of M‐DOP on the level or activity of (a) malondialdehyde (MDA), (b) glutathione (GSH), (c) superoxide dismutase (SOD), (d) catalase (CAT), and (e) glutathione peroxidase (GSH‐Px) in liver of mouse D‐galactose (D‐Gal)‐induced aging model. #*p* < .05 for the comparison of model group with control group. *p < .05 for the comparison of experimental group with model group

Analysis of liver tissues revealed greatly elevated levels of MDA, a biomarker reflecting degree of oxidation, in experimental groups relative to control group (Figure 3a), and significantly (*p* < .05) lower activities of GSH, SOD, CAT, and GSH‐Px in model group (Figure 3b–e). These findings indicate reduced capacity of antioxidant system and recovery from injury in the aging model. M‐DOP treatment effectively ameliorated oxidative stress by enhancing GSH content and SOD, CAT, and GSH‐Px activities. Reduction of MDA level reflected amelioration of lipid peroxidation in cell membrane.

### 3.5 Effect of M‐DOP treatment on hepatic pro‐inflammatory cytokine levels in a mouse D‐Gal‐induced aging model

**FIGURE 4:**
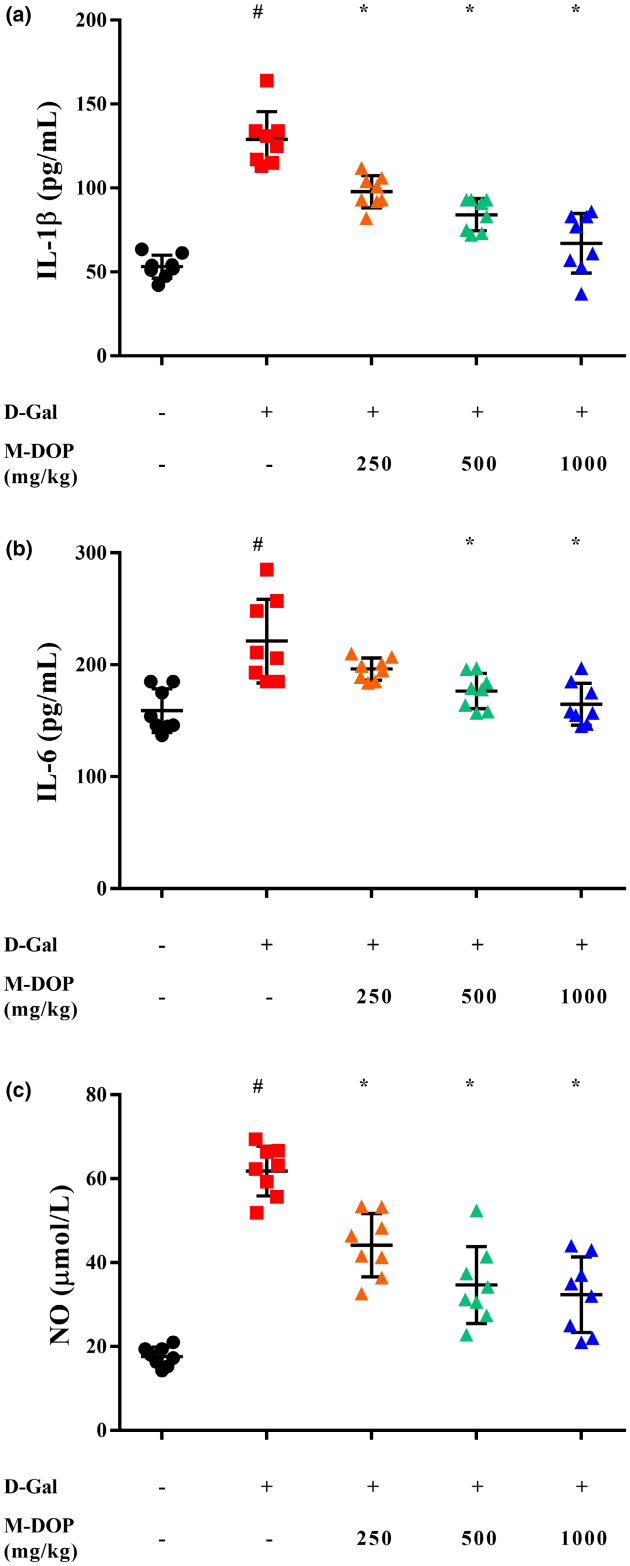
Effects of M‐DOP on the level of (a) interleukin 1β (IL‐1β), (b) interleukin 6 (IL‐6), and (c) nitric oxide (NO) in liver of mouse D‐galactose (D‐Gal)‐induced aging model. Statistical notations as in Figure 3

Reactive oxygen species function as signaling molecules in a variety of metabolic pathways in cells and tissues, including apoptotic pathways and inflammatory and developmental processes. We observed significantly higher hepatic levels of IL‐1β, IL‐6, and NO in model group than in control group (Figure 4). At the end of 8 weeks, the levels of cytokines and NO were significantly lower in M‐DOP group than in model group. NO is synthesized mainly by inducible NO synthase (iNOS) and acts as a signaling molecule in inflammatory processes. The above findings indicate that M‐DOP treatment effectively ameliorated aging‐related liver damage and suppressed inflammatory responses in our mouse model.

### 3.6 Effects of M‐DOP on expression of Nrf2, HO‐1, and NQO1

**FIGURE 5:**
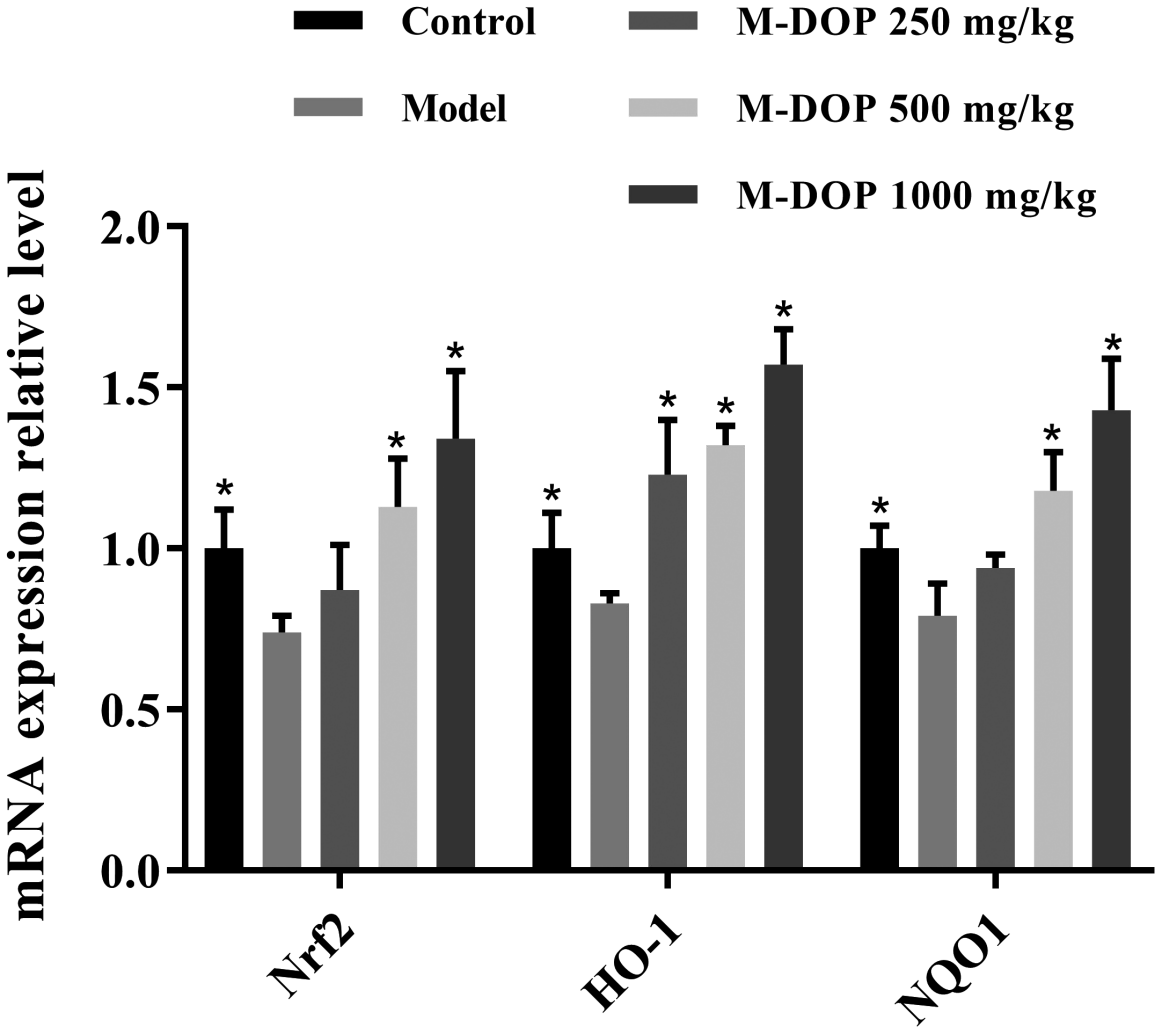
Effects of M‐DOP on messenger RNA (mRNA) expression of Nrf2 (nuclear factor erythroid 2‐related factor), heme oxygenase‐1 (HO‐1), and NAD(P)H:quinone oxidoreductase (NQO1) in liver of mouse D‐galactose (D‐Gal)‐induced aging model. *p < .05 for comparison of other groups with model group

Nrf2 (nuclear factor erythroid 2‐related factor), a redox‐sensitive transcription factor, is released from its inhibitory protein Keap1 by exposure to ROS, and triggers the expression of anti‐inflammatory/antioxidant proteins such as heme oxygenase‐1 (HO‐1) and NAD(P)H:quinone oxidoreductase 1 (NQO1). HO‐1 catalyzes the degradation of heme and various reaction products (including biliverdin, bilirubin, ferrous iron, and carbon monoxide), all of which display a strong radical scavenging activity. NQO1, a downstream antioxidant enzyme of Nrf2, reduces quinone and other oxidative stress factors. Reduced expression of pro‐inflammatory cytokines and NO caused by M‐DOP may therefore involve an Nrf2/HO‐1/NQO1 signaling pathway. Messenger RNA (mRNA) expression levels of these three proteins in liver of model (D‐Gal‐treated) group were significantly lower than in control group (Figure 5). Transcription levels of the three proteins in liver were notably enhanced by M‐DOP treatment. Increase of M‐DOP dose from 250 to 1000 mg/kg was associated with corresponding increase of mRNA levels of the three genes. These findings suggest that the antioxidant and anti‐inflammatory effects of M‐DOP in our mouse aging model depend on the Nrf2/HO‐1/NQO1 signaling pathway.

## 4 CONCLUSIONS

Ultrasonic treatment of DOP enhanced its antioxidant activity in vitro, without any change of primary structure. The increase of antioxidant activity was due primarily to reduced Mw and altered chemical composition of DOP. Among several ultrasound‐treated DOPs, M‐DOP showed the highest ROS and RNS radical scavenging activity. M‐DOP antioxidant activity in vitro was correlated with enhanced in vivo levels of biomarkers, anti‐inflammatory cytokines, and antioxidant activity in our mouse D‐Gal‐induced aging model. M‐DOP treatment ameliorated oxidative stress and inflammatory responses in the mouse model via the Nrf2/HO‐1/NQO1 signaling pathway. The methods and findings described here provide a useful basis for future commercial applications of ultrasonication, particularly in food industries.

## CONFLICT OF INTEREST

The authors declare that no competing interests were involved in the research or preparation of the manuscript.

## Supporting information

App S1

## References

[fsn32867-bib-0001] Agarwal, A. , Gupta, S. , & Sharma, R. (2005). Oxidative stress and its implications in female infertility – a clinician's perspective. Reproductive BioMedicine Online, 11(5), 641–650. 10.1016/S1472-6483(10)61174-1 16409717

[fsn32867-bib-0002] Aydın, S. , Yanar, K. , Atukeren, P. , Dalo, E. , Sitar, M. E. , Uslu, E. , Caf, N. , & Çakatay, U. (2011). Comparison of oxidative stress biomarkers in renal tissues of D‐galactose induced, naturally aged and young rats. Biogerontology, 13, 251–260. 10.1007/s10522-011-9370-3 22179795

[fsn32867-bib-0003] Bradford, M. M. (1976). Rapid and sensitive method for quantitation of microgram quantities of protein utilizing principle of protein‐dye binding. Analytical Biochemistry, 72(1–2), 248–254. 10.1006/abio.1976.9999 942051

[fsn32867-bib-0004] Byun, K. , Yoo, Y. C. , Son, M. , Lee, J. , Jeong, G.‐B. , Park, Y. M. , Salekdeh, G. H. , & Lee, B. (2017). Advanced glycation end‐products produced systemically and by macrophages: A common contributor to inflammation and degenerative diseases. Pharmacology & Therapeutics, 177, 44–55. 10.1016/j.pharmthera.2017.02.030 28223234

[fsn32867-bib-0005] Chen, B. , Zhong, Y. , Peng, W. , Sun, Y. , & Kong, W. J. (2010). Age‐related changes in the central auditory system: Comparison of d‐galactose‐induced aging rats and naturally aging rats. Brain Research, 1344, 43–53. 10.1016/j.brainres.2010.04.082 20470764

[fsn32867-bib-0006] Chen, P. , Lei, J. , Chen, F. , & Zhou, B. (2020). Ameliorative effect of urolithin A on D‐gal‐induced liver and kidney damage in aging mice via its antioxidative, anti‐inflammatory and antiapoptotic properties. RSC Advances, 10(14), 8027–8038. 10.1039/d0ra00774a 35497859 PMC9049876

[fsn32867-bib-0007] Çoban, J. , Doğan‐Ekici, I. , Aydin, A. F. , Betül‐Kalaz, E. , Doğru‐Abbasoğlu, S. , & Uysal, M. (2015). Blueberry treatment decreased D‐galactose‐induced oxidative stress and brain damage in rats. Metabolic Brain Disease, 30(3), 793–802. 10.1007/s11011-014-9643-z 25511550

[fsn32867-bib-0008] Dodgson, K. S. , & Price, R. G. (1962). A note on determination of ester sulphate content of sulphated polysaccharides. Biochemical Journal, 84(1), 106–110. 10.1042/bj0840106 13886865 PMC1243628

[fsn32867-bib-0009] Dubois, M. , Gilles, K. A. , Hamilton, J. K. , Rebers, P. A. , & Smith, F. (1956). Colorimetric method for determination of sugars and related substances. Analytical Chemistry, 28(3), 350–356. 10.1021/ac60111a017

[fsn32867-bib-0010] Fan, L. M. , & Li, J.‐M. (2014). Evaluation of methods of detecting cell reactive oxygen species production for drug screening and cell cycle studies. Journal of Pharmacological and Toxicological Methods, 70(1), 40–47. 10.1016/j.vascn.2014.03.173 24721421

[fsn32867-bib-0011] He, L. , Yan, X. , Liang, J. , Li, S. , He, H. , Xiong, Q. , Lai, X. , Hou, S. , & Huang, S. (2018). Comparison of different extraction methods for polysaccharides from *Dendrobium officinale* stem. Carbohydrate Polymers, 198, 101–108. 10.1016/j.carbpol.2018.06.073 30092979

[fsn32867-bib-0012] Hipkiss, A. R. , Preston, J. E. , Himsworth, D. M. , Worthington, V. C. , Keown, M. , Michaelis, J. , Lawrence, J. , Mateen, A. , Allende, L. , Eagles, P. M. , & Abbott, N. J. (1998). Pluripotent protective effects of carnosine, a naturally occurring dipeptide. Annals of the New York Academy of Sciences, 854, 37–53. 10.1111/j.1749-6632.1998.tb09890.x 9928418

[fsn32867-bib-0013] Hou, Y. , Wang, J. , Jin, W. H. , Zhang, H. , & Zhang, Q. B. (2012). Degradation of *Laminaria japonica* fucoidan by hydrogen peroxide and antioxidant activities of the degradation products of different molecular weights. Carbohydrate Polymers, 87(1), 153–159. 10.1016/j.carbpol.2011.07.031 34662944

[fsn32867-bib-0014] Huang, K. W. , Li, Y. R. , Tao, S. C. , Wei, G. , Huang, Y. C. , Chen, D. F. , & Wu, C. F. (2016). Purification, characterization and biological activity of polysaccharides from *Dendrobium officinale* . Molecules, 21(6), 701. 10.3390/molecules21060701 PMC6272863 27248989

[fsn32867-bib-0015] Li, D.‐D. , Li, W.‐J. , Kong, S.‐Z. , Li, S.‐D. , Guo, J.‐Q. , Guo, M.‐H. , Cai, T.‐T. , Li, N. , Chen, R.‐Z. , Luo, R.‐Q. , & Tan, W.‐X. (2019). Protective effects of collagen polypeptide from tilapia skin against injuries to the liver and kidneys of mice induced by D‐galactose. Biomedicine & Pharmacotherapy, 117, 109204. 10.1016/j.biopha.2019.109204 31387177

[fsn32867-bib-0016] Li, H. Z. , Pordesimo, L. , & Weiss, J. (2004). High intensity ultrasound‐assisted extraction of oil from soybeans. Food Research International, 37(7), 731–738. 10.1016/j.foodres.2004.02.016

[fsn32867-bib-0017] Li, L. , Xu, M. , Shen, B. , Li, M. , Gao, Q. , & Wei, S. G. (2016). Moderate exercise prevents neurodegeneration in D‐galactose‐induced aging mice. Neural Regeneration Research, 11(5), 807–815. 10.4103/1673-5374.182709 27335566 PMC4904473

[fsn32867-bib-0018] Liu, J. , Pu, Q. , Qiu, H. , & Di, D. (2021). Polysaccharides isolated from *Lyciumbarbarum* L. by integrated tandem hybrid membrane technology exert antioxidant activities in mitochondria. Industrial Crops and Products, 168, 113547. 10.1016/j.indcrop.2021.113547

[fsn32867-bib-0019] Mzoughi, Z. , Chakroun, I. , Ben Hamida, S. , Rihouey, C. , Ben Mansour, H. , Le Cerf, D. , & Majdoub, H. (2017). Ozone treatment of polysaccharides from *Arthrocnemum indicum*: Physico‐chemical characterization and antiproliferative activity. International Journal of Biological Macromolecules, 105, 1315–1323. 10.1016/j.ijbiomac.2017.07.151 28756195

[fsn32867-bib-0020] Naik, E. , & Dixit, V. M. (2011). Mitochondrial reactive oxygen species drive proinflammatory cytokine production. Journal of Experimental Medicine, 208(3), 417–420. 10.1084/jem.20110367 21357740 PMC3058577

[fsn32867-bib-0021] Palma‐Duran, S. A. , Kontogianni, M. D. , Vlassopoulos, A. , Zhao, S. , Margariti, A. , Georgoulis, M. , Papatheodoridis, G. , & Combet, E. (2018). Serum levels of advanced glycation end‐products (AGEs) and the decoy soluble receptor for AGEs (sRAGE) can identify non‐alcoholic fatty liver disease in age‐, sex‐ and BMI‐matched normo‐glycemic adults. Metabolism, 83, 120–127. 10.1016/j.metabol.2018.01.023 29409822

[fsn32867-bib-0022] Pan, L.‐H. , Li, X.‐F. , Wang, M.‐N. , Zha, X.‐Q. , Yang, X.‐F. , Liu, Z.‐J. , Luo, Y.‐B. , & Luo, J.‐P. (2014). Comparison of hypoglycemic and antioxidative effects of polysaccharides from four different *Dendrobium* species. International Journal of Biological Macromolecules, 64, 420–427. 10.1016/j.ijbiomac.2013.12.024 24370475

[fsn32867-bib-0023] Peng, L. , Qiao, S. , Xu, Z. , Guan, F. , Ding, Z. , Gu, Z. , Zhang, L. , & Shi, G. (2015). Effects of culture conditions on monosaccharide composition of *Ganoderma lucidum* exopolysaccharide and on activities of related enzymes. Carbohydrate Polymers, 133, 104–109. 10.1016/j.carbpol.2015.07.014 26344261

[fsn32867-bib-0024] Qiu, J. Q. , Zhang, H. , & Wang, Z. Y. (2019). Ultrasonic degradation of polysaccharides from *Auricularia auricula* and the antioxidant activity of their degradation products. Lwt‐Food Science and Technology, 113, 108266. 10.1016/j.lwt.2019.108266

[fsn32867-bib-0025] Saleh, D. , Mansour, D. , Hashad, I. , & Bakeer, R. (2019). Effects of sulforaphane on D‐galactose‐induced liver aging in rats: Role of keap‐1/nrf‐2 pathway. European Journal of Pharmacology, 855, 40–49. 10.1016/j.ejphar.2019.04.043 31039346

[fsn32867-bib-0026] Salmon, A. B. , Richardson, A. , & Pérez, V. I. (2010). Update on the oxidative stress theory of aging: Does oxidative stress play a role in aging or healthy aging? Free Radical Biology and Medicine, 48(5), 642–655. 10.1016/j.freeradbiomed.2009.12.015 20036736 PMC2819595

[fsn32867-bib-0027] Salzano, S. , Checconi, P. , Hanschmann, E.‐M. , Lillig, C. H. , Bowler, L. D. , Chan, P. , Vaudry, D. , Mengozzi, M. , Coppo, L. , Sacre, S. , Atkuri, K. R. , Sahaf, B. , Herzenberg, L. A. , Herzenberg, L. A. , Mullen, L. , & Ghezzi, P. (2014). Linkage of inflammation and oxidative stress via release of glutathionylated peroxiredoxin‐2, which acts as a danger signal. Proceedings of the National Academy of Sciences of the United States of America, 111(33), 12157–12162. 10.1073/pnas.1401712111 25097261 PMC4143057

[fsn32867-bib-0028] Wang, B. , Li, L. , Chi, C. F. , Ma, J. H. , Luo, H. Y. , & Xu, Y. F. (2013). Purification and characterisation of a novel antioxidant peptide derived from blue mussel *(Mytilus edulis*) protein hydrolysate. Food Chemistry, 138(2–3), 1713–1719. 10.1016/j.foodchem.2012.12.002 23411302

[fsn32867-bib-0029] Wang, C. , Gao, Y. , Tao, Y. , Wu, X. Z. , & Cui, Z. B. (2017). gamma‐Irradiation treatment decreased degradation of cell‐wall polysaccharides in blueberry fruit during cold storage. Postharvest Biology and Technology, 131, 31–38. 10.1016/j.postharvbio.2017.04.012

[fsn32867-bib-0030] Xing, X. , Cui, S. W. , Nie, S. , Phillips, G. O. , Douglas Goff, H. , & Wang, Q. (2013). A review of isolation process, structural characteristics, and bioactivities of water‐soluble polysaccharides from *Dendrobium*plants. Bioactive Carbohydrates and Dietary Fibre, 1(2), 131–147. 10.1016/j.bcdf.2013.04.001

[fsn32867-bib-0031] Yan, J. K. , Li, L. , Wang, Z. M. , Leung, P. H. , Wang, W. Q. , & Wu, J. Y. (2009). Acidic degradation and enhanced antioxidant activities of exopolysaccharides from *Cordyceps sinensis* mycelial culture. Food Chemistry, 117(4), 641–646. 10.1016/j.foodchem.2009.04.068

[fsn32867-bib-0032] Yan, J. K. , Wang, Y. Y. , Ma, H. L. , & Wang, Z. B. (2016). Ultrasonic effects on the degradation kinetics, preliminary characterization and antioxidant activities of polysaccharides from *Phellinus linteus* mycelia. Ultrasonics Sonochemistry, 29, 251–257. 10.1016/j.ultsonch.2015.10.005 26585005

[fsn32867-bib-0033] Yan, S. , Pan, C. , Yang, X. , Chen, S. , Qi, B. , & Huang, H. (2021). Degradation of *Codium cylindricum* polysaccharides by H_2_O_2_‐Vc‐ultrasonic and H_2_O_2_‐Fe^2+^‐ultrasonic treatment: Structural characterization and antioxidant activity. International Journal of Biological Macromolecules, 182, 129–135. 10.1016/j.ijbiomac.2021.03.193 33831452

[fsn32867-bib-0034] Yan, X. , Wang, W. W. , Liu, M. J. , & Zhao, Z. H. (2018). Preparation of oligosaccharides by degradation of polysaccharides from Chinese jujube and its biological activity. International Journal of Polymer Science, 2018, 1–8. 10.1155/2018/6464051

[fsn32867-bib-0035] Yang, L. Y. , Xian, D. H. , Xiong, X. , Lai, R. , Song, J. , & Zhong, J. Q. (2018). Proanthocyanidins against oxidative stress: From molecular mechanisms to clinical applications. Biomed Research International, 2018, 1–11. 10.1155/2018/8584136 PMC5884402 29750172

[fsn32867-bib-0036] Yao, Y. , Zhu, Y. Y. , Gao, Y. , & Ren, G. X. (2015). Effect of ultrasonic treatment on immunological activities of polysaccharides from adlay. International Journal of Biological Macromolecules, 80, 246–252. 10.1016/j.ijbiomac.2015.06.033 26123819

[fsn32867-bib-0037] Yen, G. C. , Lai, H. H. , & Chou, H. Y. (2001). Nitric oxide‐scavenging and antioxidant effects of *Urariacrinita* root. Food Chemistry, 74(4), 471–478. 10.1016/s0308-8146(01)00165-0

[fsn32867-bib-0038] Yu, G. , Zhao, J. , Wei, Y. , Huang, L. , Li, F. , Zhang, Y. , & Li, Q. (2021). Physicochemical properties and antioxidant activity of pumpkin polysaccharide (*Cucurbita moschata Duchesne* ex Poiret) modified by subcritical water. Foods, 10(1), 197. 10.3390/foods10010197 33478048 PMC7835828

[fsn32867-bib-0039] Zhou, Y.‐Y. , Ji, X.‐F. , Fu, J.‐P. , Zhu, X.‐J. , Li, R.‐H. , Mu, C.‐K. , Wang, C.‐L. , & Song, W.‐W. (2015). Gene transcriptional and metabolic profile changes in mimetic aging mice induced by D‐Galactose. PLoS One, 10, e0132088. 10.1371/journal.pone.0132088 26176541 PMC4503422

[fsn32867-bib-0040] Zhu, Z.‐Y. , Pang, W. , Li, Y.‐Y. , Ge, X.‐R. , Chen, L.‐J. , Liu, X.‐C. , Lv, Q. , Dong, G.‐L. , Liu, A.‐J. , & Zhang, Y. (2014). Effect of ultrasonic treatment on structure and antitumor activity of mycelial polysaccharides from *Cordyceps gunnii* . Carbohydrate Polymers, 114, 12–20. 10.1016/j.carbpol.2014.07.068 25263858

