# Supplementary material for: Ultrasonic treatment of Dendrobium officinale polysaccharide enhances antioxidant and anti‐inflammatory activity in a mouse D‐galactose‐induced aging model
Source: Food Sci Nutr. 2022 Apr 1;10(8):2620–30. doi: 10.1002/fsn3.2867 (PMC9361453; doi:10.1002/fsn3.2867)
Supplement: Supplementary file 1 — App S1 [file FSN3-10-2620-s001.docx]

# Supplementary materials

Figure S1. Intrinsic viscosity changes of DOP subjected to ultrasonic treatment at various power intensities.


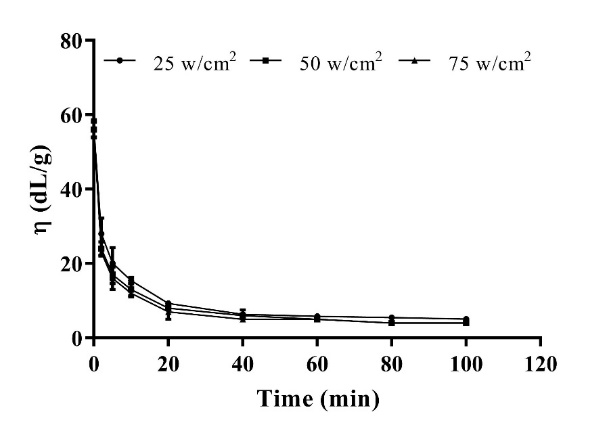


Figure S2. Infrared spectra of DOP subjected to ultrasonic treatment at various power intensities. N-DOP: no ultrasonic treatment, L-DOP: 25 w/cm^2^, M-DOP: 50 w/cm^2^, H-DOP: 75 w/cm^2^.


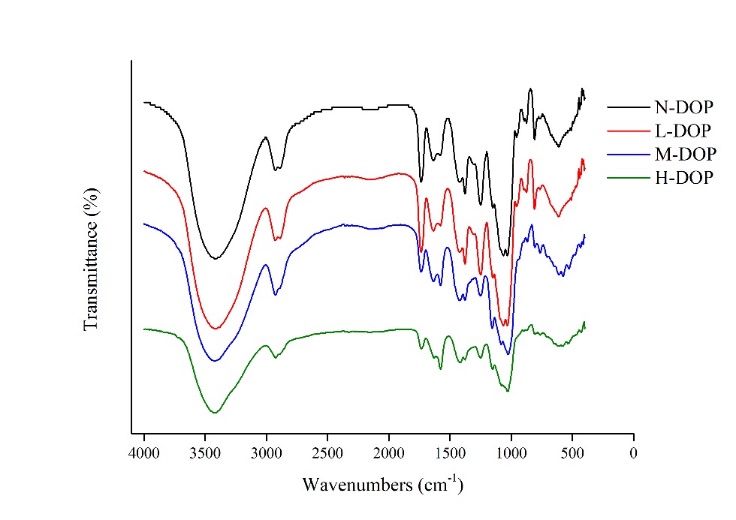


Figure S3. Effects of low (250 mg/kg BW/day), medium (500 mg/kg BW/day), and high (1000 mg/kg BW/day) dose of M-DOP on BW changes of mice during 8-wk feeding.


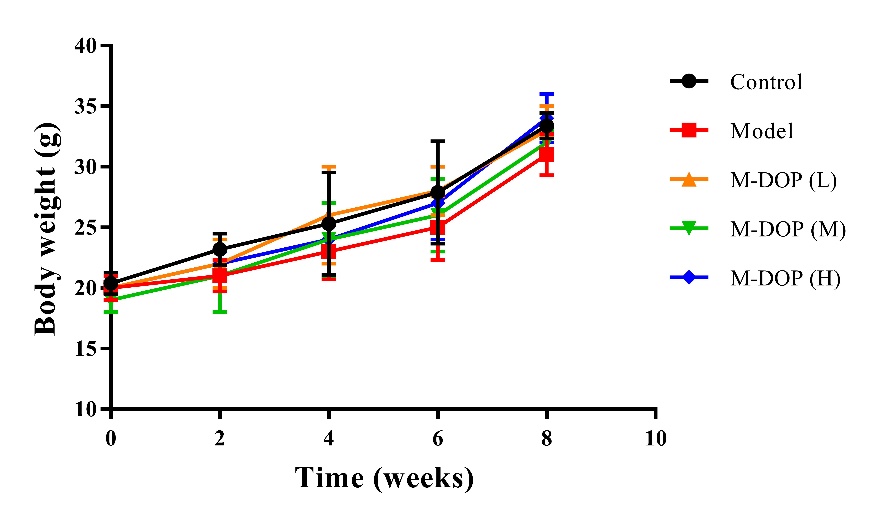


Figure S4. Effects of M-DOP on serum activity or level of **(A)** ALT, **(B)** ASP, **(C)** ALP, and **(D)** CREA in mouse D-Gal-induced aging model. Statistical notations as in Fig. 3.


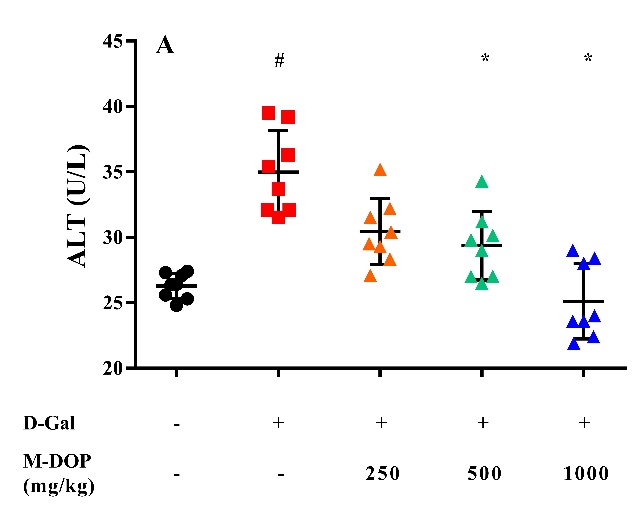


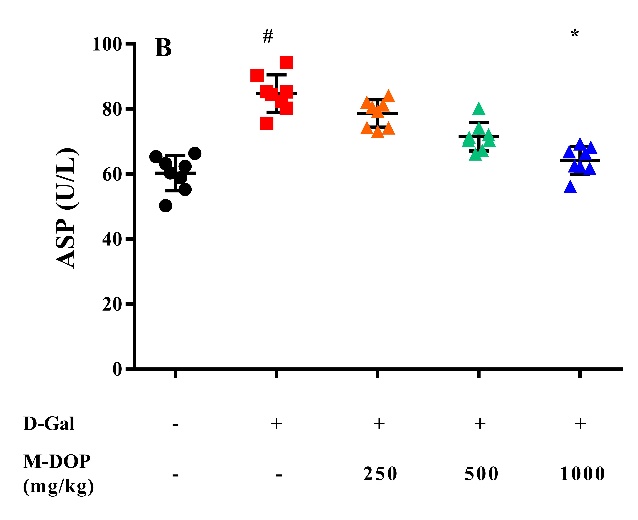


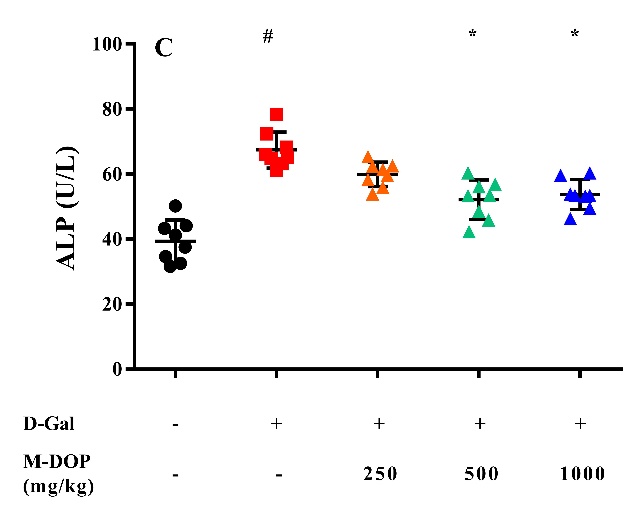


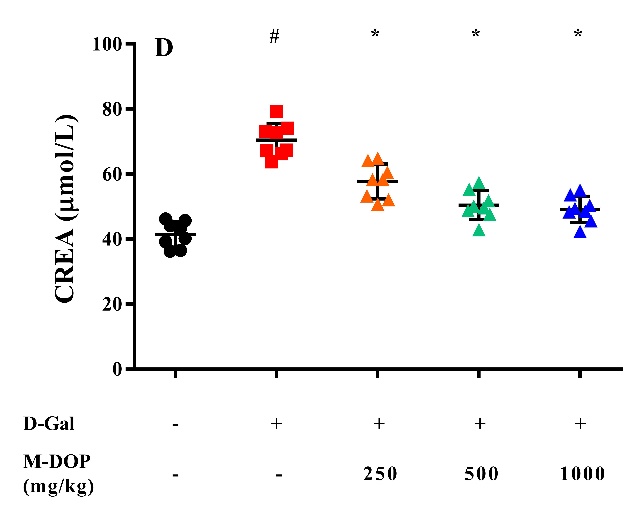


Table S1. Primer sequences for RT-PCR.

| Gene | Primer sequences |
| --- | --- |
| HO-1 | forward: 5’GAGTGGGGCATAGACTGGGTT3’  reverse: 5’GCTGGTGATGGCTTCCTTGTA3’ |
| Nrf2 | forward: 5’GAAAAAGAAGTGGGCAACTGTGG-3’  reverse: 5’GGTGGGATTTGAGTCTAAGGAGGT-3’ |
| NQO1 | forward: 5’GGTATTACGATCCTCCCTCAACATC3’  reverse: 5’GGTATTACGATCCTCCCTCAACATC3’ |
| GAPDH | forward: 5’AGGTCGGTGTGAACGGATTTG3’  reverse: 5’GGGGTCGTTGATGGCAACA3’ |

Table S2. Effect of ultrasonic treatment at various power intensities on chemical composition of DOP.

| DOP | Carbohydrates (%) | Proteins (%) | Sulfate radicals (%) |
| --- | --- | --- | --- |
| N-DOP | 86.52 ± 1.24^c^ | 3.68 ± 0.25^a^ | 4.43 ± 0.3^a^ |
| L-DOP (60 min) | 90.48 ±1.27^b^ | 1.44 ±0.16^ab^ | 3.33 ± 0.10^a^ |
| M-DOP (60 min) | 93.09 ±0.82^ab^ | 1.03 ± 0.13a^bc^ | 1.52 ± 0.05^b^ |
| H-DOP (60 min) | 94.29 ± 0.91^a^ | 0.61 ± 0.11^c^ | 0.64 ± 0.08^c^ |

Notations as in Table. 1. Data shown are mean ± SD (n=3).
